# Gastric *Helicobacter* Infection Induces Iron Deficiency in the INS-GAS Mouse

**DOI:** 10.1371/journal.pone.0050194

**Published:** 2012-11-19

**Authors:** Melanie J. Thomson, D. Mark Pritchard, Sally A. Boxall, Abdul A. Abuderman, Jonathan M. Williams, Andrea Varro, Jean E. Crabtree

**Affiliations:** 1 Molecular Gastroenterology, Leeds Institute of Molecular Medicine, St. James’s University Hospital, Leeds, United Kingdom; 2 Institute of Translational Medicine, University of Liverpool, Liverpool, United Kingdom; Veterans Affairs Medical Center (111D), United States of America

## Abstract

There is increasing evidence from clinical and population studies for a role of *H. pylori* infection in the aetiology of iron deficiency. Rodent models of *Helicobacter* infection are helpful for investigating any causal links and mechanisms of iron deficiency in the host. The aim of this study was to investigate the effects of gastric *Helicobacter* infection on iron deficiency and host iron metabolism/transport gene expression in hypergastrinemic INS-GAS mice. INS-GAS mice were infected with *Helicobacter felis* for 3, 6 and 9 months. At post mortem, blood was taken for assessment of iron status and gastric mucosa for pathology, immunohistology and analysis of gene expression. Chronic *Helicobacter* infection of INS- GAS mice resulted in decreased serum iron, transferrin saturation and hypoferritinemia and increased Total iron binding capacity (TIBC). Decreased serum iron concentrations were associated with a concomitant reduction in the number of parietal cells, strengthening the association between hypochlorhydria and gastric *Helicobacter*-induced iron deficiency. Infection with *H. felis* for nine months was associated with decreased gastric expression of iron metabolism regulators hepcidin, *Bmp4* and *Bmp6* but increased expression of Ferroportin 1, the iron efflux protein, iron absorption genes such as Divalent metal transporter 1, Transferrin receptor 1 and also *Lcn2* a siderophore-binding protein. The INS-GAS mouse is therefore a useful model for studying *Helicobacter*-induced iron deficiency. Furthermore, the marked changes in expression of gastric iron transporters following *Helicobacter* infection may be relevant to the more rapid development of carcinogenesis in the *Helicobacter* infected INS-GAS model.

## Introduction

Iron deficiency is the most common nutritional disorder in the world with iron deficiency anemia (IDA) affecting 500–600 million people globally (WHO). Manifestations of clinically advanced IDA include increased childhood mortality, reduced growth, cognitive function and susceptibility to infectious diseases [1]. Accumulating evidence from clinical [2], [3], [4], [5], [6] and population [7], [8], [9], [10], [11] studies implicates gastric *Helicobacter pylori* infection in the aetiology of iron deficiency and IDA. Iron deficiency in *H. pylori* infection is resistant to iron supplementation, but is reversible by bacterial eradication [5], [7], [12], [13], [14], [15]. *H. pylori*-associated IDA is particularly prevalent in children, who have smaller iron stores compared with adults [16].

There are several mechanisms by which *H. pylori* might contribute to iron deficiency and IDA. These include competition between the pathogen and host for limited iron stores [13], [17], [18], particularly in individuals with iron poor diets, thus exacerbating the effects of reduced iron intake. Iron is essential for *H. pylori* growth and survival, but this pathogen does not produce siderophores conventionally used by other bacteria to acquire iron [19]. The ability of *H. pylori* to sequester iron facilitates its colonisation of the acidic gastric environment via enhanced protection from oxidative stress [20]. Inactivation of iron transport and storage proteins in *H. pylori* renders it incapable of host colonisation. *H. pylori* iron binding proteins have also been implicated in the aetiology of IDA [18], [21]. Hypochlorhydria associated with acute *H. pylori* infections may predispose to IDA due to the inability to generate the bioavailable, reduced form of iron (ferrous, Fe^2+^) under non-acidic conditions [22], [23], [24]. Iron-limiting conditions induce expression of recognised virulence factors in *H. pylori*, including the Cytotoxin associated gene A (CagA) and the vacuolating toxin A (VacA) which damage epithelial cells, thus enhancing the ability of *H. pylori* to acquire iron released from damaged host cells [25], [26]. *H. pylori* can bind and extract iron from haemoglobin, transferrin, and lactoferrin [27]. Unlike other pathogens, *H. pylori* preferentially binds iron-free forms of transferrin and lactoferrin, limiting host extraction of iron. In a polarised *in vitro* epithelial cell model, host iron trafficking is perturbed by the aberrant localisation of the transferrin receptor to the apical surface instead of the basolateral surface of the epithelium following CagA and VacA positive *H. pylori* infection [28]. In gerbils on an iron deficient diet, *ΔcagA* strains have impaired colonisation, suggesting that CagA has a role in pathogen iron acquisition *in vivo* [28]. The effect of *H. pylori* infection and dietary iron deficiency on host iron homeostasis has been studied in wild type C57BL/6 mice [29], [30]. *H. pylori* diminished further iron stores, measured by serum ferritin and liver iron, in mice on an iron deficient diet [30]. Gastric *Helicobacter felis*, but not *H. pylori* infection, caused iron deficiency assessed by serum iron and ferritin levels in non pregnant and pregnant mice [29]. Taken together, these and other studies provide evidence that iron acquisition by *H. pylori* may have a significant role in the development of IDA.

The transgenic INS-GAS mouse model over-expresses the human gastrin (hGAS) gene controlled by the rat insulin promoter, resulting in hypergastrinemia. Male INS-GAS mice on the FVB/N background spontaneously develop gastric adenocarcinoma after 20 months of age and this process is accelerated to 8 months by gastric *Helicobacter* infection [31]. A positive correlation between circulating gastrin and transferrin saturation has been seen in hypergastrinemic patients [32]. Gastrin may play a direct role in modulating iron homeostasis as hepatic transcripts of the iron regulator hepcidin (*Hamp*) were increased in Gastrin-resistant CCK2 receptor knockout (CCK2RKO) mice, which have increased levels of circulating gastrin and decreased in gastrin knockout (GasKO) mice maintained on an iron deficient diet [33]. The aim of this study was therefore to investigate the haematological and molecular changes relating to iron deficiency resulting from gastric *Helicobacter* infection in hypergastrinemic INS-GAS mice. This was achieved by using a series of clinically relevant iron assays combined with molecular investigations of the expression of host iron metabolism and absorption genes in the gastric mucosa. As with the earlier studies from Wang *et al* [31], we have used *H. felis* infection rather than *H. pylori* as our earlier studies have demonstrated this cause greater inflammation than *H. pylori* in the murine model [35].

## Methods

### Ethics Statement

All experimental procedures and breeding of the INS-GAS transgenic mice were undertaken at the University of Liverpool according to UK Home Office guidelines. The study was undertaken on project licence number PPL 40/2833 which was approved by the local ethics committee of the University of Liverpool and the UK Home Office. The welfare of the mice was monitored daily.

### Bacteria

*Helicobacter felis* ATCC 49179 was cultured as previously described [46] at 37°C for 2–3 days in a microaerophilic atmosphere. Bacteria were harvested and suspended in Tryptone Soya Broth (TSB, Oxoid) to a density of approx 5×10^8^ CFU/ml and were used immediately.

### H. felis Infection of Mice

Male transgenic INS-GAS mice on a FVB/N genetic background, kindly supplied by Professor Timothy C. Wang (Columbia University, New York, USA), were bred at the University of Liverpool conventional animal facility. Male FVB/N mice were purchased from Harlan UK Ltd, (Bichester, UK). INS-GAS mice (*Helicobacter* species free) at 6–8 weeks of age were inoculated three times by oral gavage with *H. felis* (>10^8^ CFU) suspended in tryptone soya broth (Oxoid) over a period of 1 week. Infected mice (n = 16−19 per group) and uninfected controls (n = 19−20 per group) were sacrificed at 3, 6 and 9 months post-inoculation.

Following euthanasia with CO_2_, whole blood was collected via cardiac puncture; serum and plasma were stored at −20°C. The stomach was removed, incised along the greater curvature and rinsed with phosphate buffered saline. Gastric corpus tissue from the 9 month cohort was stored in ‘RNA Later’ (Invitrogen) at −20°C prior to total RNA extraction. Antral and corpus tissue were fixed in 4% formal saline and embedded in paraffin wax for histological analysis.

For histological assessment of parietal cell numbers the whole stomach was dissected from the mouse. Ligatures were tied around the distal oesophagus and proximal duodenum, and the stomach lumen was infiltrated with 4% formal saline. This procedure distended the stomach and fixed gastric glands in a reliable manner for histological assessment. Tissue was paraffin embedded and sections were cut at a thickness of 4 µm. Histology and *H. felis* infection were assessed on Haematoxylin and Eosin and Giemsa stained sections. Gastric histopathology was assessed in a blind, randomised fashion by a Veterinary Pathologist (JMW) and graded according to the scoring system of Rogers *et al*. [36].

### Immunohistology

Immunohistochemistry was used to detect and quantify parietal cells. Following permeabilization by incubating dewaxed and rehydrated sections with 1% Triton × in phosphate buffered saline for 45 minutes at room temperature, sections were incubated overnight at 4°C in rabbit anti-rat H^+^/K^+^ ATPase polyclonal antibody (1∶1000, Calbiochem) diluted in 10% goat serum in Tris buffered saline (TBS) with 1% bovine serum albumin (BSA). Sections were washed in TBS-Tween20 and incubated with HRP-labelled anti-rabbit antibody (Envision kit (Dako UK Ltd) for 30 minutes before washing and substrate development according to the manufacturer’s instructions. Sections were counterstained with Gill’s haematoxylin. To quantify cell numbers in the gastric corpus mucosa, ten areas were chosen from each section and H^+^-K^+^-ATPase-positive cells were counted using a Leica light microscope.

### Iron Parameters

Serum iron concentration and unsaturated iron binding capacity (UIBC) were measured directly using a microtitre plate assay based on change in absorbance on addition of Ferrozine (Sigma) according to the standard protocols in Practical Haematology [37]. Total Iron binding capacity (TIBC) was calculated from Serum Iron+UIBC. Transferrin saturation was calculated by Serum Iron/TIBC × 100. Taken together, these three parameters form an important part of the routine diagnostic assessment for iron deficiency anaemia and anaemia of chronic disease in clinical settings.

### Serum Ferritin

Serum ferritin protein concentrations were measured by a mouse specific enzyme-linked immunosorbent assay (ELISA) supplied by Kamiya Biochemical Company (Seattle, WA, USA) according to the manufacturer’s instructions. Clinically, the assessment of circulating ferritin levels is used as a surrogate marker for stored iron in the host.

### Real Time PCR

Total RNA was extracted by homogenising gastric tissue using the TRIzol® Reagent (Invitrogen) according to the manufacturer’s instructions. Superscript II (Invitrogen) was used to create cDNA, according to the manufacturer’s instructions. Quantitative real time PCR was performed using standard techniques on an ABI 7500 machine, using SYBR® green dye (Applied Biosystems). The expression of target genes was assessed as relative abundance compared to *Gapdh* control gene by the ΔΔCt method [38]. Primer sequences are listed in Table 1 and were designed to cross an intron/exon boundary or taken from published literature [39].

**Table 1:** Real time PCR primers.

Locus	Name^a^	Nucleotide Sequence	Reference
Total Hepcidin	*HampF*	5′-AGAGCTGCAGCCTTTGCAC-3′	This study
	*HampR*	5′-GAGGTCAGGATGTGGCTCTA-3′	
Ferroportin 1	*Fpn1F*	5′-TTGCAGGAGTCATTGCTGCTA-3′	This study
	*Fpn1R*	5′-GGAGTTCTGCACACCATTGAT-3′	
Bone Morphogenic Protein 4	*Bmp4F*	5′-TGAGTACCCGGAGCGTCC-3′	Kautz *et al* 2008
	*Bmp4R*	5′-CTCCAGATGTTCTTCGTGATGG-3′	
Bone Morphogenic Protein 6	*Bmp6F*	5′-ATGGCAGGACTGGATCATTGC-3′	Kautz *et al* 2008
	*Bmp6R*	5′-CCATCACAGTAGTTGGCAGCG-3′	
Divalent metal transporter 1	*Dmt1F*	5′-GGCTTTCTTATGAGCATTGCCTA-3′	This study
	*Dmt1R*	5′-GGAGCACCCAGAGCAGCTTA-3′	
Transferrin receptor 1	*Tfr1F*	5′-CATGAGGGAAATCAATGATCGTA-3′	This study
	*Tfr1R*	5′-GCCCCAGAAGATATGTCGGAA-3′	
Lipocalin 2	*Lcn2F*	5′-CTGAATGGGTGGTGAGTGTG-3′	This study
	*Lcn2R*	5′-GCTCTCTGGCAACAGGAAAG-3′	

### Statistics

Statistical analysis was performed using GraphPad PRISM software, utilising Student’s *t* test (unpaired with Welch’s correction) or Mann-Whitney U test as appropriate after assessing distribution of data with the Kruskal-Wallis test. Data were considered significant if p≤0.05.

## Results

### Effects of H. felis Infection on INS-GAS Iron Parameters

#### Serum iron concentration decreases in chronically *Helicobacter* infected INS-GAS mice

**Figure 1:**
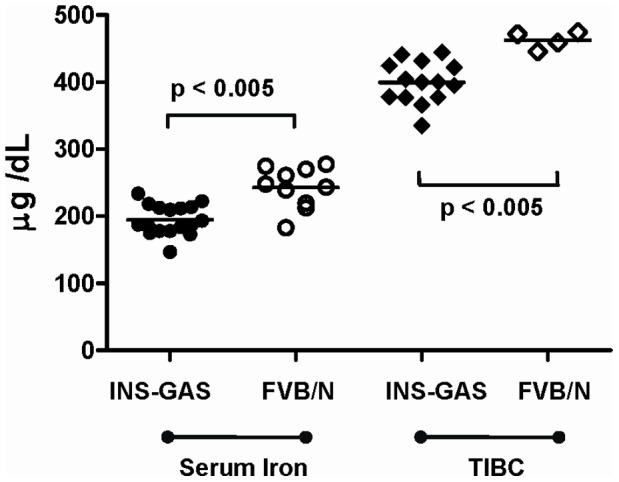
Iron parameters in INS-GAS and FVB/N mice at 11 months of age. Scatter plots of individual serum iron concentrations (**circles**) and total iron binding capacity (TIBC) (**diamonds**) in transgenic INS-GAS mice (**solid markers**) (n = 17) and genetic age and gender-matched FVB/N control mice (**open markers**) (iron, n = 10; TIBC, n = 4). Insufficient serum was available for TIBC analysis in 6 FVB/N control mice. Statistical analysis by Mann-Whitney U test.

**Figure 2:**
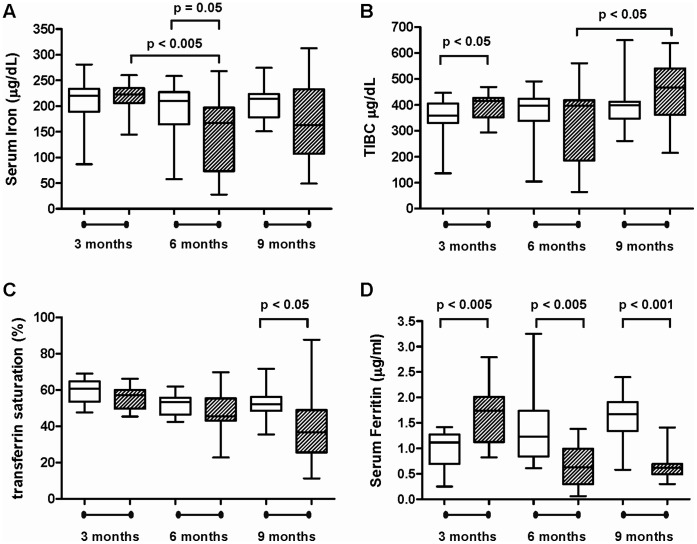
Iron parameters in INS-GAS mice at 3, 6 and 9 months post-infection with *H. felis.* Box and whisker plots of serum iron (**A**), total iron binding capacity (TIBC) (**B**), transferrin saturation (**C**) and serum ferritin (**D**) in uninfected (**white boxes**) and *H. felis* infected (**hatched boxes**) INS-GAS mice. Time points as marked, group size n = 16−20, whiskers represent maximum/minimum values. Statistical analysis by unpaired *t* test with Welch’s correction.

The serum iron assay measures the amount of circulating iron that is bound to transferrin, one of the host iron carriers. Initial studies showed that uninfected INS-GAS mice have no difference in transferrin saturation (data not shown) but a significantly reduced concentration of serum iron (p<0.005) and TIBC (p<0.005) compared to uninfected FVB/N mice at 10-11 months age (Figure 1). Although some bias may have been introduced due to the purchased FVB/N control mice being obtained from a different breeding colony to background strain used to produce the INS-GAS mice, this is still evidence that there is a reduction in iron parameters in this transgenic line, making it a suitable model for further investigation of *Helicobacter*-induced iron deficiency. In uninfected INS-GAS mice there was no significant change in serum iron concentrations at 3, 6 or 9 months (Figure 2a, white boxes). In contrast, at 6 months, serum iron concentrations in *H. felis* infected mice were significantly lower (p<0.005) than in 3 month *H. felis* infected mice, and also significantly reduced (p = 0.05) relative to 6 month uninfected controls (Figure 2a). No further reduction in serum iron concentration was evident at 9 months in *H. felis* infected mice (Figure 2a).

#### Total iron binding capacity increases over time in *Helicobacter* infected INS-GAS mice

The total iron-binding capacity (TIBC) assay saturates the sample to measure the total amount of transferrin in circulation. At 3 months TIBC in *H. felis* infected mice was significantly increased (p<0.05) compared to controls (Figure 2b). TIBC in infected mice was further increased (p<0.05) at 9 months compared to 6 month infected mice (Figure 2b). In uninfected INS-GAS mice there was no significant change in TIBC at 3, 6 or 9 months (Figure 2b, white boxes).

#### Transferrin saturation is reduced in chronically *Helicobacter* infected INS-GAS mice

The transferrin saturation assay measures the extent to which sites on transferrin molecules are occupied by iron ions. The percentage of transferrin saturation was also decreased in infected mice, becoming significantly reduced (p<0.05) by 9 months post-infection compared to control mice (Figure 2c). No age related changes were evident in uninfected INS-GAS mice.

#### Serum Ferritin increases then decreases over time in *Helicobacter* infected INS-GAS mice

Ferritin is a ubiquitous intracellular protein that sequesters iron and releases it in a controlled manner. The amount of circulating serum is used clinically as a surrogate biomarker for decreased iron stores in the host. In the early phase (3 months) of the *H. felis* infection, serum ferritin concentration was significantly increased compared to uninfected mice (p<0.005, Figure 2d). In contrast, at the later stages of the infection, serum ferritin concentrations were significantly reduced (Figure 2d, p<0.005 at 6 months; p<0.001 at 9 months), perhaps reflecting the overall lack of iron absorbed by the host due to the hypochlorhydria associated with parietal cell loss.

### Reduced Parietal Cell Numbers in INS-GAS Mice with Gastric Helicobacter Infection

**Figure 3:**
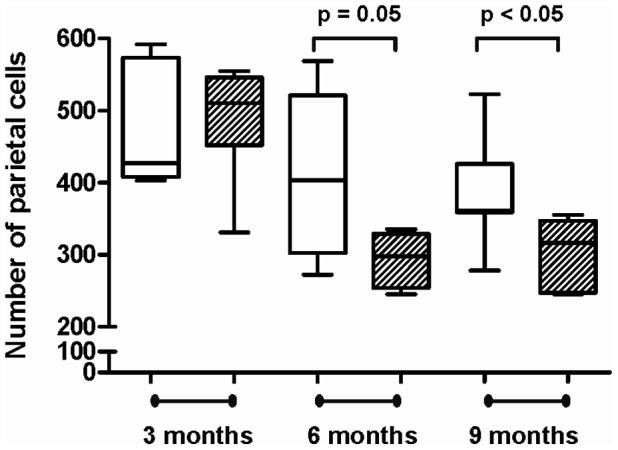
Parietal cell number in INS-GAS mice at 3, 6 and 9 months post-infection with *H. felis.* Box and whisker plots of gastric parietal cell number in uninfected (**white boxes**) and *H. felis* infected (**hatched boxes**) INS-GAS mice. Group size n = 5−7 mice, whiskers represent maximum/minimum values. Statistical test is an unpaired *t* test with Welch’s correction.

As gastrin may have a direct role in modulating iron homeostasis [32], [34] and the hypochlorhydria associated with *H. pylori-*induced corpus atrophy is associated with iron deficiency [22], gastric parietal numbers were assessed in the INS-GAS mice and the relationship between iron status and the plasma gastrin levels previously determined in the mice was assessed. There was no significant correlation between iron concentrations and plasma gastrin concentrations in this cohort (data not shown). The decrease in serum iron concentrations at 6 months in *H. felis* infected mice however was associated with a concomitant decrease in gastric parietal cell numbers relative to controls (p = 0.05), which was also evident at 9 months (p<0.05) (Figure 3).

### Effects of H. felis Infection on INS-GAS Gastric Mucosa

**Figure 4:**
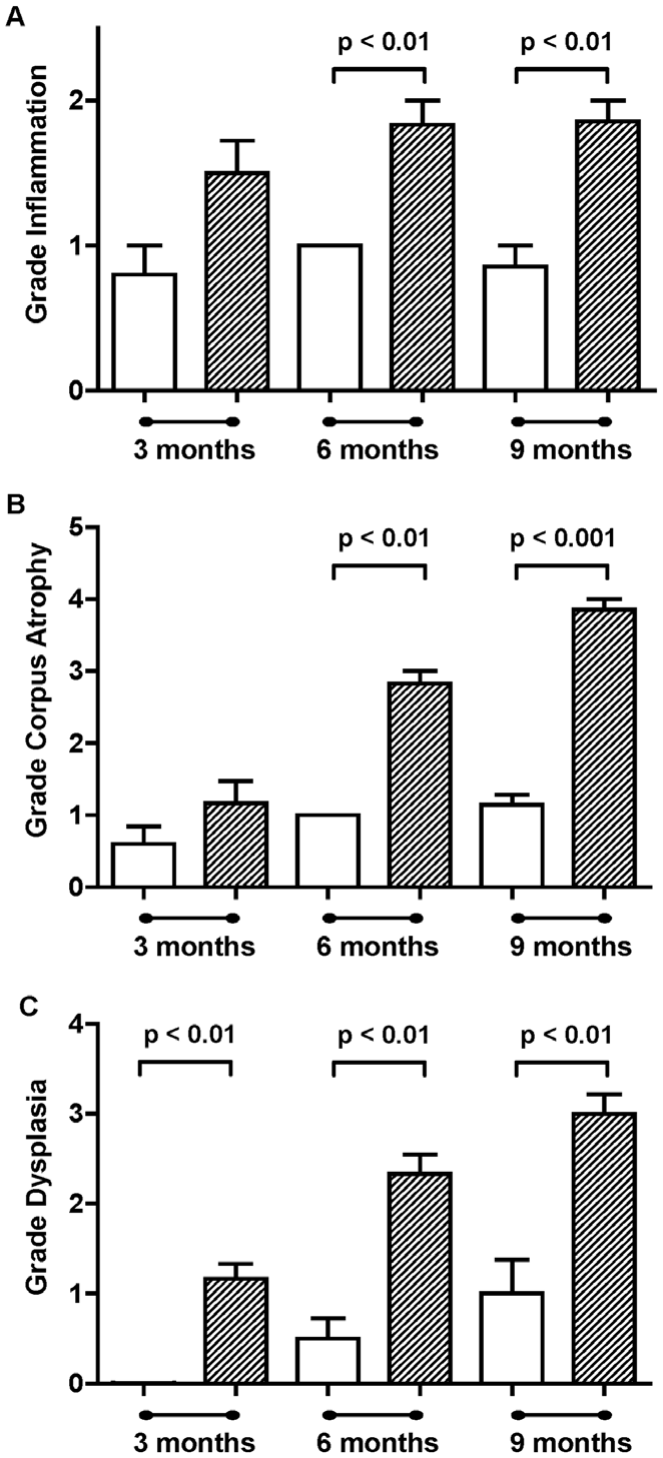
Gastric histopathology in INS-GAS mice at 3,6 and 9 months post-infection with *H. felis*. Grade of gastric inflammation (**A**), corpus atrophy (**B**) and dysplasia (**C**) in uninfected control (**white bars**) and *H. felis* infected INS-GAS mice (**hatched bars**). Group size n = 5–7. Error bars represent mean ± SEM, when error present. Statistical analysis by the Mann Whitney U test.

All *H. felis* inoculated mice with available pathology at 3 and 6 months were histologically positive for the bacterium. At 9 months, *H. felis* could be detected in 55% of the inoculated animals, however, all mice showed marked inflammation and mucosal hyperplasia, indicative of successful infection. Loss of gastric *Helicobacter* infection with corpus atrophy has been frequently document both clinically and in rodent models. *Helicobacter felis* infected INS-GAS mice exhibited increasing severity of gastric mucosal lesions proportional with the duration of infection (Figure 4). Lesions were characterised by mucosal hyperplasia, loss of parietal cells and chief cells (oxyntic gland atrophy), mucous metaplasia, dilated gastric glands, and inflammatory infiltrates consisting predominantly of lymphocytes, plasma cells, and to a lesser extent neutrophils. Gastric glands of longer term infected animals often exhibited features of dysplasia with disorganised branching, cell crowding with loss of polarity, hyperchromatic enlarged nuclei and increased mitotic figures.

Significantly increased inflammation (p<0.01) was evident at both 6 months and 9 months in *H. felis* infected INS-GAS mice compared to INS-GAS control mice (Figure 4a). Oxyntic gland atrophy in *H. felis* infected INS-GAS mice increased progressively with time being significantly greater than controls at both 6 months (p<0.01) and 9 months post-infection (p<0.001) (Figure 4b). In contrast, in the control INS-GAS mice no significant increase in inflammation was observed over the 9 months and only a small increase in oxyntic gland atrophy at 6 and 9 months was observed. None of these changes were statistically significant from the 3 month controls. A significant increase (p<0.01) in gastric dysplasia was evident in *H. felis* infected INS-GAS mice compared to INS-GAS control mice (Figure 4c) consistent with earlier studies [31]. No significant differences were observed temporarily between the control groups.

### Effects of H. felis Infection on INS-GAS Iron Metabolism Genes

#### Chronic H. felis infection reduces the expression of the iron metabolism regulator, Hepcidin, in the gastric mucosa

**Figure 5:**
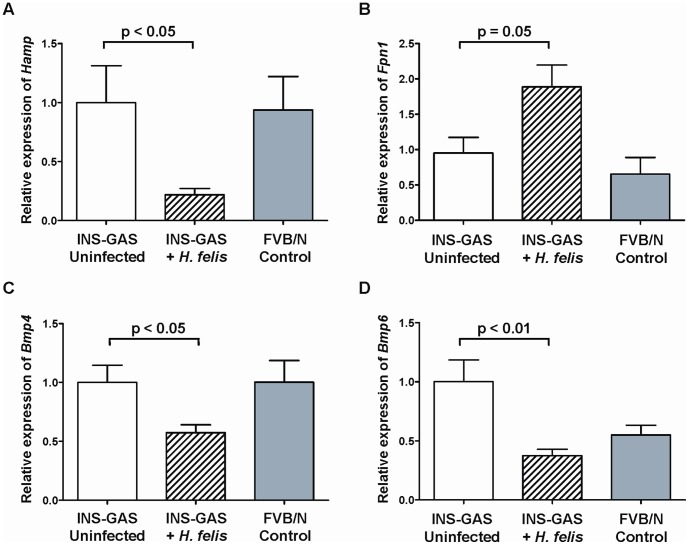
Expression of iron metabolism regulation genes in murine gastric mucosa. Relative expression levels of genes involved in the regulation of iron metabolism were measured by qRT-PCR in biopsies of the gastric corpus mucosa of INS-GAS mice at 9 months post-infection with *H. felis* and in age-matched uninfected INS-GAS mice and FVB/N controls. The graphs represent the mRNA abundance of the test genes relative to the house keeping gene, *Gapdh*. Uninfected (**white bars**) (n = 10) and *H. felis* infected (**hatched bars**) transgenic INS-GAS mice (n = 10) were compared to the uninfected FVB/N genetic background control (**solid bars**) (n = 5). The relative levels of total hepcidin genes (*Hamp 1* and *2*) (**A**), Ferroportin 1 (*Fpn1*) gene (**B**), bone morphogenic protein gene 4 (*Bmp4*) (**C**) and 6 (*Bmp6*) gene (**D**) were assessed. Error bars represent mean ± SEM. Statistical analysis by unpaired *t* test with Welch’s correction.

Hepcidin is a hormone that controls the levels of iron in the labile iron pool for use in iron metabolism by negatively regulating the expression of Ferroportin 1 on iron trafficking cells and modulating the absorption of iron from ingested food via the small intestine. Hepcidin expression is regulated by IL6 in response to inflammation. A significant increase (p<0.01) in the abundance of gastric corpus *Il6* transcripts was observed in the INS-GAS mice compared to the FVB/N controls but no significant difference (p = 0.57) was observed between the *H. felis* infected and uninfected control mice (data not shown). Again, it is possible that the significant difference between the INS-GAS transgenic mice and the FVB/N control mice could be due to a possible bias introduced to the experimental system, as they originate from different breeding colonies. INS-GAS mice infected with *H. felis* for 9 months (Figure 5a, hatched bar) showed a significant reduction in hepcidin transcripts in the gastric mucosa compared to both the uninfected INS-GAS and FVB/N mice. The abundance of transcripts for the upstream regulators of Hepcidin, *Bmp4* (p<0.05, Figure 5c) and *Bmp6* (p<0.01, Figure 5d) were also significantly reduced following 9 months of *H. felis* infection in INS-GAS gastric corpus mucosa. Gastric corpus *Bmp6* expression in FVB/N control mice was lower but not significantly different (p = 0.06) than uninfected INS-GAS mice.

Ferroportin 1 is an efflux protein that is expressed on the basolateral membranes of gastrointestinal cells. This protein is negatively regulated by hepcidin, which binds to Ferroportin1 on the surface of cells inducing degradation. A significant increase (p = 0.05) in the abundance of the corpus transcripts of *Fpn1* was observed in the INS-GAS mice 9 months following *H. felis* infection (Figure 5b, hatched bar). Gastric corpus *Fpn1* expression in FVB/N control mice was slightly lower (p = 0.28) than uninfected INS-GAS mice.

#### Chronic *H. felis* infection increases the expression of iron absorption genes in the gastric mucosa

**Figure 6:**
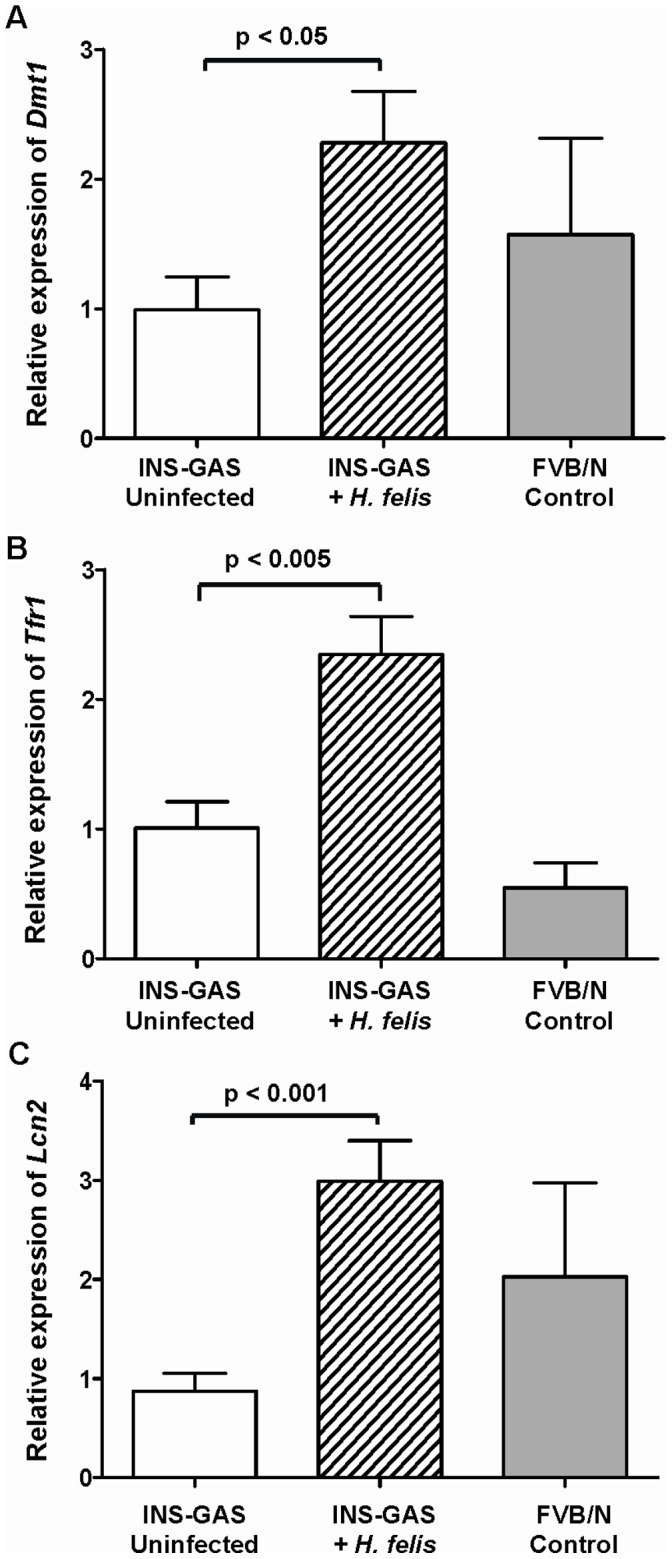
Expression of iron binding and transport genes in murine gastric mucosa. Relative expression levels of genes involved in the binding and transport of iron were measured by qRT-PCR in tissue taken from the gastric corpus mucosa of INS-GAS mice at 9 months post-infection with *H. felis* and in age-matched uninfected INS-GAS mice and FVB/N controls. The graphs represent the mRNA abundance of the test genes relative to the house keeping gene, *Gapdh*. Uninfected (**white bars**) (n = 10) and *H. felis* infected (**hatched bars**) transgenic INS-GAS mice (n = 10) were compared to the uninfected FVB/N genetic background control (**solid bars**) (n = 5). The relative levels of Divalent Metal transporter 1 gene (*Dmt1*) (**A**), Transferrin receptor 1 (*Tfr1*) gene (**B**) and Lipocalin 2 (*Lcn2*) gene (**C**) were assessed. Error bars represent mean ± SEM. Statistical analysis by unpaired *t* test with Welch’s correction.

Divalent metal transporter 1 (DMT1), is expressed on the apical membrane of gastrointestinal cells and absorbs reduced ferrous iron (Fe^2+^) from the gut lumen. There was a significant increase (p<0.05) in relative *Dmt1* transcripts in the gastric corpus mucosa of INS-GAS mice infected with *H. felis* for 9 months (Figure 6a, hatched bar) compared to uninfected controls. Gastric corpus *DMT1* expression in FVB/N control mice was higher but not significantly different (p = 1.0) from uninfected INS-GAS mice.

Transferrin receptor 1 (TFR1) is a protein that is widely expressed on mammalian tissues. It binds and internalises (via endocytosis) circulating iron-loaded transferrin. Hepatocytes, which modulate hepcidin concentrations, sense the saturation of transferrin (via an unknown mechanism) and release the regulator hepcidin in response. *Tfr1* transcripts were significantly up-regulated in the gastric mucosa of *H. felis* infected INS-GAS mice at 9 months (Figure 6b, hatched bar, p<0.005). Gastric corpus *Tfr1* expression in FVB/N control mice was lower but not significantly different (p = 0.20) from uninfected INS-GAS mice.

Lipocalin 2 (Lcn2, also known as ***Neutrophil gelatinase-associated lipocalin*** [NGAL] or Holo-24p3) is a host outer membrane protein that binds to bacterial siderophores loaded with iron and is thought to bind an endogenous host equivalent (yet to be identified). In *H. felis* infected INS-GAS mice, there was a significant increase (Figure 6c, hatched bar, p<0.001) in *Lcn2* transcripts compared to the uninfected INS-GAS control cohort at 9 months. This shows the inverse relationship between hepcidin transcripts (low in infected group) and the increased expression of a gene whose product is associated with host iron metabolism. Gastric corpus *Lcn2* expression in FVB/N control mice was higher (not significantly, p = 0.32) than uninfected INS-GAS mice.

## Discussion

The aim of this study was to use the INS-GAS mouse model to explore the hematological and molecular changes relating to iron deficiency resulting from gastric *Helicobacter* infection. INS-GAS mice with chronic gastric *Helicobacter* infection have significantly raised circulating amidated gastrin concentrations compared to uninfected INS-GAS controls at 9 months post infection [40]. This increase is over and above the existing elevated concentrations observed in uninfected INS-GAS mice compared to their FVB/N genetic background controls. These data concur with previous observations resulting from gastric *Helicobacter* infection in the INS-GAS model [41]. Conversely, parietal cell numbers in *H. felis* infected INS-GAS mice were reduced significantly at 6 and 9 months post-inoculation concomitant with the decrease in serum iron concentrations. The reduction in the number of parietal cells and associated hypochlorhydria induced by gastric *Helicobacter* infection may lead to poor ferrous iron (Fe^2+^) absorption. Gastric acid is important for reducing dietary ferric (Fe^3+^) iron to ferrous (Fe^2+^) iron. In the achlorhydric Atp4a^−/−^ mouse, which has a mutation in the 4a subunit of H^+^/K^+^ ATPase, the failure of iron absorption is considered to be the combined effects of poor solubility of ferric iron and impaired Dmt1 function due to the reduced levels of protons in the stomach [42].

The haematological parameters at the chronic stages of *Helicobacter* infection in the present study are indicative of poor iron absorption. At 6 months post-infection, serum iron concentrations were significantly lower due to *H. felis* infection and by 9 months post-infection, the transferrin saturation was also markedly lower in the infected cohort. This reflects the systemic iron deficiency of these animals when compared to their uninfected counterparts, as they have reduced circulating iron loaded onto the transferrin protein. Iron storage capability, as measured by serum ferritin, increased in the acute phase of the infection at 3 months, as the host attempted to sequester iron so that it that it could not be used by the pathogen. However, by the chronic stage of the infection (6 and 9 months post-inoculation) serum ferritin concentrations were significantly reduced, perhaps in response to the overall lack of systemic iron, rendering the requirement for iron storage obsolete. Keenan *et al* observed a similar pattern of raised serum ferritin concentrations at 10 weeks post-inoculation followed by a reduction at 30 weeks after *H. pylori* infection of non-transgenic mice fed on an iron-restricted diet [30].

In this study, *H. felis* infection modified the abundance of gastric mucosal mRNA of various iron metabolism genes. Some of these genes, such as *Fpn1, Dmt1* and *Tfr1* are regulated post-transcriptionally and future studies are required confirm relative protein levels. Immunohistological studies confirmed Dmt1 protein was increased in gastric epithelial cells in *H. felis* infection (data not shown).

Hepcidin is one of the major regulators of iron homeostasis in the host and due to its reported antimicrobial activity, is often expressed in epithelial tissues that form the first line of defence against bacterial colonisation, such as the lungs and the glandular stomach [43]. Hence it is not surprising that ample *Hamp* mRNA was found in the gastric corpus mucosa of uninfected INS-GAS and FVB/N mice in this study. A recent study using hepcidin knockout mice [44] has shown that hepcidin can regulate acid secretion and vice versa. The absence of hepcidin resulted in a rise in gastric pH that was accompanied by bacterial overgrowth. In the present study, *Hamp* mRNA transcripts were significantly reduced in the gastric mucosa of *H. felis* infected INS-GAS mice when compared to both uninfected INS-GAS mice and FVB/N controls. This may be due to the significant reduction in the number of parietal cells, which have recently been shown to be the site of hepcidin expression in the gastric mucosa [44]. The same study examined the effect of *H. pylori* infection on hepcidin expression using the human AGS epithelial cell *in vitro* model and found that hepcidin expression increased in response to inflammation in this system. This was reflected in parallel studies using human gastric tissue where, in contrast to the present study, increased hepcidin expression was observed in the antral and corpus mucosa in the presence of *H. pylori* infection [44]. In humans, *H. pylori* infection is not associated with an increase in serum hepcidin [44] or urinary hepcidin [45] suggesting that gastric *Helicobacter*–induced changes in hepcidin do not have a systemic correlate.

The effects of the decrease in gastric hepcidin expression in infected INS-GAS mice on the expression of various downstream iron absorption and efflux genes in the gastric mucosa were examined. Ferroportin 1, an iron efflux protein, is normally degraded after interacting with hepcidin. Consistent with this, we observed a significant concomitant increase in gastric *Fpn1* transcripts which is likely to result directly from the reduced localised hepcidin. The transcripts of other iron binding and transport genes such as *Dmt1* and *Tfr1* were also significantly up-regulated in an indirect response to the decreased hepcidin levels in the 9 month *H. felis* infected INS-GAS mice. This is likely to be due to the decrease in cytosolic iron which is detected by the iron responsive transcriptional regulators IRP1 and 2; these in turn activate the expression of *Dmt1* and *Tfr1* genes. These data, combined with the fluctuation of the serum ferritin levels, reflect a shift in the host response from iron storage in the acute phase of the infection, to iron acquisition in the latter stages of infection. Despite this apparent increase in the host’s ability to absorb, release and transport iron, the chronically infected INS-GAS mice had poor iron status as measured by transferrin saturation and serum ferritin. The majority of iron absorption will be in the small intestine and the functional importance of hepcidin and iron transporters in the gastric mucosa remains to be investigated. Gastric and small intestinal expression of proteins involved in iron metabolism and absorption may be differentially regulated. It is well established that iron trafficking proteins in the duodenum control the rate of iron absorption, impacting on systemic iron parameters [46]. The functional importance of iron metabolism proteins in the gastric mucosa and related changes to systemic iron parameters associated with gastric *Helicobacter* infection, remains to be investigated.

Lipocalin 2 protein expression has been previously shown to increase in humans with gastritis in response to *H. pylori* infection [47], [48]. This is thought to be related to the function of Lcn2 as a siderophore-binding protein and hence an innate host response to restrict bio-available iron from invading pathogens [49]. *H. pylori* does not produce siderophores in order to harvest iron from its environment [19] and hence is intrinsically resistant to this form of host defence. Lipocalin 2 may have evolved in the host to bind iron-rich cathecholamines (such as dopamine and epinephrine) described as ‘mammalian siderophores’ [50]. It is intriguing to speculate that *Helicobacter* species can in some way exploit this localised iron supply as they have siderophore-uptake outer membrane proteins (such as TonB, FeoB) even though they do not produce these molecules [51]. Strains of *H. pylori* with polymorphisms in the *feoB* gene have been related to subsequent impaired iron status in a human population in Korea, although this study was statistically under-powered [18].

Previous studies have shown that expression of proteins from the bone morphogenic pathway (such as BMP4) in the human gastric mucosa can be altered by *H. pylori* infection [52], [53]. Members of the BMP pathway (such as BMP6 and SMAD) also positively control hepcidin gene expression. The gastric expression of *Bmp4* and *Bmp6* in INS-GAS mice was significantly decreased following *H. felis* infection. The three fold decrease in *Bmp6* expression correlated with the decrease in hepcidin expression observed. This is interesting as the source of hepcidin in the gastric mucosa is said to be the parietal cells [44], but the expression of *Bmp* genes is part of the stem cell signalling network and is hence likely to occur in the stem cell compartment. Our results are supported by several other studies that have implicated *H. pylori* in the dysregulation of these cells [54], [55], [56].

In conclusion, chronic gastric *Helicobacter* infection in INS-GAS mice results in decreased serum iron concentrations, hypoferritinemia and transferrin saturation and increased TIBC. Decreased serum iron concentrations were associated with a concomitant reduction in the number of parietal cells, strengthening the association between hypochlorhydria and gastric *Helicobacter*-induced iron deficiency. Infection with *H. felis* for nine months was associated with decreased gastric expression of iron metabolism regulators such as hepcidin, *Bmp4* and *Bmp6* but increased expression of ferroportin 1, the iron efflux protein, and iron absorption genes such as *Dmt1*, Transferrin receptor 1 and also *Lcn2* a siderophore-binding protein. The INS-GAS murine model is therefore a useful model for studying *Helicobacter*-induced iron deficiency. Furthermore, the marked changes in gastric iron transporters with *Helicobacter* infection identified in this study may be relevant to the more rapid development of carcinogenesis in INS-GAS mice following *Helicobacter* infection.
